# Rrp6 Regulates Heterochromatic Gene Silencing via ncRNA RUF6 Decay in Malaria Parasites

**DOI:** 10.1128/mBio.01110-20

**Published:** 2020-06-02

**Authors:** Yanting Fan, Shijun Shen, Guiying Wei, Jianxia Tang, Yuemeng Zhao, Fei Wang, Xiaohui He, Gangqiang Guo, Xiaomin Shang, Xinyu Yu, Zhenlin Ma, Xiaoqin He, Meng Liu, Qianshu Zhu, Zhen Le, Gang Wei, Jun Cao, Cizhong Jiang, Qingfeng Zhang

**Affiliations:** a Research Center for Translational Medicine, Key Laboratory of Arrhythmias of the Ministry of Education of China, East Hospital, Tongji University School of Medicine, Shanghai, China; b Institute of Translational Research, Tongji Hospital, the School of Life Sciences and Technology, Shanghai Key Laboratory of Signaling and Disease Research, Tongji University, Shanghai, China; c National Health Commission Key Laboratory of Parasitic Disease Control and Prevention, Jiangsu Provincial Key Laboratory on Parasite and Vector Control Technology, Jiangsu Institute of Parasitic Diseases, Wuxi, China; d CAS Key Laboratory of Computational Biology, CAS-MPG Partner Institute for Computational Biology, Shanghai Institute of Nutrition and Health, Shanghai Institutes for Biological Sciences, University of Chinese Academy of Sciences, Chinese Academy of Sciences, Shanghai, China; e Center for Global Health, School of Public Health, Nanjing Medical University, Nanjing, China; f Public Health Research Center, Jiangnan University, Wuxi, China; g The Research Center of Stem Cells and Ageing, Tsingtao Advanced Research Institute, Tongji University, Tsingtao, China; NIAID/NIH

**Keywords:** RNA exosome, malaria, heterochromatin, gene regulation, ncRNA

## Abstract

The heterochromatin environment plays a central role in silencing genes associated with the malaria parasite’s development, survival in the host, and transmission to the mosquito vector. However, the underlying mechanism regulating the dynamic chromatin structure is not understood yet. Here, we have uncovered that Plasmodium falciparum Rrp6, an orthologue of eukaryotic RNA exosome-associated RNase, controls the silencing of heterochromatic genes. PfRrp6 knockdown disrupted the singular expression of the GC-rich ncRNA RUF6 family, a known critical regulator of virulence gene expression, through the stabilization of the nascent transcripts. Mechanistic investigation showed that the accumulation of the multiple RUF6 ncRNAs triggered local chromatin remodeling *in situ*, which activated their adjacent *var* genes. Strikingly, chromatin isolation by RNA purification analysis (ChIRP-seq) revealed that a remarkable RUF6 ncRNA had interacted with distal heterochromatin regions directly and stimulated a global derepression effect on heterochromatic genes, including all variant gene families and the sexual commitment-associated regulator *ap2-g* gene. Collectively, Rrp6 appears to conduct the epigenetic surveillance of heterochromatic gene expression through controlling RUF6 levels, thereby securing antigenic variation and sexual commitment of malaria parasites during the infection of the host.

## Abstract (precis)

Malaria remains a major public health and economic burden. The heterochromatin environment controls the silencing of genes associated with the fate of malaria parasites. Previous studies have demonstrated that a group of GC-rich ncRNAs (RUF6) is associated with the mutually exclusive expression of *var* genes, but the underlying mechanisms remain elusive. Here, through a series of genetic manipulation and genome-wide multiomics analysis, we have identified the plasmodial orthologue of RNA exosome-associated Rrp6 as an upstream regulator of RUF6 expression and revealed that the dysregulation of RUF6 upon Rrp6 knockdown triggered local chromatin alteration, thereby activating most heterochromatic genes via direct interaction of RUF6 and distal gene loci. This finding not only uncovered the in-depth mechanism of RUF6-mediated regulation of heterochromatic genes but also identified Rrp6 as a novel regulator of gene expression in human malaria parasites, which provides a new target for developing intervention strategies against malaria.

## INTRODUCTION

Malaria, caused by the unicellular protozoan parasite of genus *Plasmodium*, accounts for approximately two hundred million clinical cases and 405,000 deaths annually (1). Malaria parasites harbor complex life cycles in human and mosquito vectors, and the tight regulation of the gene expression program determines multiple physiological processes of development, infection, and pathogenesis in the host (2–4). In eukaryotes, the highly condensed heterochromatin structure is generally associated with gene silencing (5). In the most severe form of malaria parasites, Plasmodium falciparum, the heterochromatin protein 1 (HP1)-mediated heterochromatic environment controls the transcriptional silencing of genes, such as clonally variant genes, invasion genes, and the *ap2-g* gene, which are involved in antigenic variation, red blood cell (RBC) invasion, and sexual commitment, respectively (6–8).

The sophisticated expression mechanism of a single member of the ∼60 members of the *var* gene family, encoding the P. falciparum erythrocyte membrane protein 1 (PfEMP1), is one of the most successful examples of the regulatory function of heterochromatin (9, 10). The highly ordered nuclear heterochromatin structure marked by HP1 and the histone modification H3K9me3 fulfilled the mutually exclusive expression of the *var* gene family by silencing most members by default, whereas the single active member located in the euchromatin microenvironment is marked by H3K9ac and H3K4me2/3 in a specialized zone at the nuclear periphery (6, 11–13). However, in other eukaryotic organisms, even the constitutive heterochromatin was dynamically regulated in response to stimuli (14). As for the malaria parasites, it has been reported that the local chromatin alteration was linked to the activation of subtelomeric *var* genes (9, 15), but the underlying mechanisms of heterochromatin maintenance and local chromatin remodeling during expression switching are still elusive.

Besides the regulatory pathway at the transcriptional level, a growing body of evidence revealed another layer at the posttranscriptional level. Recent studies utilizing new techniques of ATAC-seq (assay for transposase-accessible chromatin by sequencing) or nascent RNA-seq (transcriptome sequencing) discovered that local chromatin accessibility and nascent RNA abundance were more predictive of gene transcriptional activity than steady-state RNAs (16–19). Importantly, they showed that nascent transcripts were pervasive in the genome of P. falciparum. These findings suggest that posttranscriptional mechanisms execute functions comparable to those of histone modifications in the regulation of gene expression in this pathogen. As for the nascent RNA metabolism, the eukaryotic conserved RNA exosome complex is responsible for the decay of nascent transcripts immediately after their production (20, 21). Our previous study had demonstrated that PfRNase II, an exosome-independent exoribonuclease, mediated the posttranscriptional silencing of a subtype of severe malaria-associated *var* genes by nascent mRNA degradation *in situ* (22). However, it is still unknown whether there exists common exosome-mediated posttranscriptional regulation of heterochromatic genes.

In eukaryotic organisms, the RNA exosome complex is composed of nine essential core subunits and two catalytic cofactors with 3′–5′ exoribonuclease activity, Rrp44 (Dis3 in human) and Rrp6 (EXOSC10 in human) (23–25). It controls RNA processing and quality surveillance in the nucleus or cytoplasm through various pathways (20, 26, 27). In P. falciparum, an inducible gene knockout approach by the DiCre system or TRIBE analysis found that P. falciparum Dis3 (PfDis3) was involved in shaping the dynamic transcriptome for either sense or antisense transcripts, suggesting a general function of posttranscriptional regulation in this parasite (28, 29). For the other cofactor, PfRrp6 (PF3D7_1449700), its characteristics and biological functions had not yet been investigated systematically. In this study, we report, for the first time, the unique evolutionary state, biological role, and underlying mechanism of PfRrp6 in P. falciparum.

## RESULTS

### Characterization of the orthologue of Rrp6 in P. falciparum.

**FIG 1:**
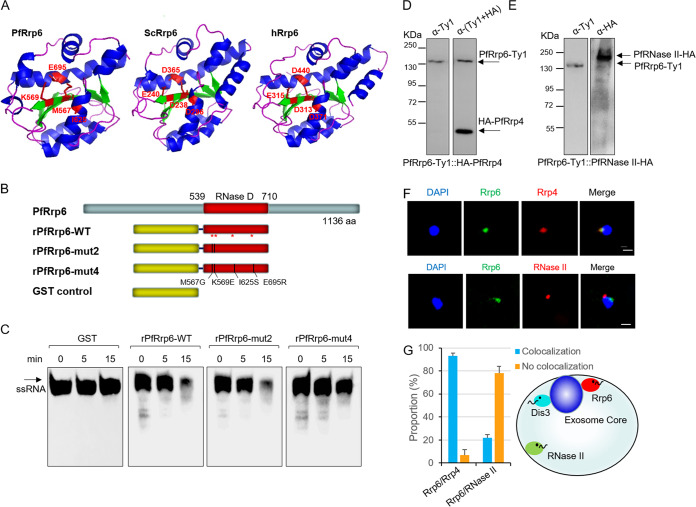
Characterization of the Rrp6 orthologue in P. falciparum. (A) Modeling of the crystal structure of the conserved catalytic RNase D domain of PfRrp6, S. cerevisiae Rrp6 (KZV07883.1), and human Rrp6 (NP_001001998.1). The catalytic residues DEDD and MKIE are indicated individually. (B) Schematic representation of three recombinant PfRrp6 variants and the glutathione *S*-transferase (GST) control. The mutated residues corresponding to MKIE are shown at the bottom. (C) Exoribonuclease assay *in vitro*. ssRNA, 17-mer ssRNA probe. (D and E) Western blot analysis of PfRrp6-Ty1::HA-PfRrp4 (left) and PfRrp6-Ty1::PfRNase II-HA (right) with antibodies against Ty1 (mouse) or HA (rabbit), respectively. The bands of target genes are indicated by arrows. (F) Co-IFA of PfRrp6 and PfRrp4 or PfRrp6 and PfRNase II with the PfRrp6-Ty1::HA-PfRrp4 or PfRrp6-Ty1::PfRNase II-HA line, respectively. DAPI, 4′,6-diamidino-2-phenylindole. (G, left) The bar graph represents the percentage of colocalization or noncolocalization of PfRrp6-PfRrp4 or PfRrp6-PfRNase II, respectively. For each experiment, ∼50 nuclei were examined. Error bars represent standard errors of the means (SEM) for three biological replicates. ***, *P < *0.001 (χ^2^ test). (G, right) A schematic illustration of the subcellular distribution of RNA exosome-associated ribonucleases (Rrp6 and Dis3) and PfRNase II.

First, to evaluate the evolutionary role of Rrp6 protein in eukaryotes, including malaria parasite species, we constructed a phylogenetic tree with the sequences of the catalytic RNase D domain superfamily of Rrp6 orthologues. It revealed that Rrp6 proteins in the *Plasmodium* genus actually formed a unique clade by themselves (see Fig. S1A in the supplemental material), but, intriguingly, the highly conserved DEDD catalytic residues (30) were not present in any species of malaria parasites (Fig. S1B). However, we did not know whether such residue variation would influence the enzymatic activity. To this end, we performed crystal structure prediction and enzymatic activity analysis *in vitro*. Structural modeling revealed a conserved catalytic structure of the RNase D domains of Rrp6 from human, yeast, and P. falciparum. In particular, the four putative catalytic residues in PfRrp6, MKIE, formed an enzymatic space similar to that of DEDD of human or yeast Rrp6 (Fig. 1A). To examine if the putative catalytic residues (MKIE) are responsible for the exoribonuclease activity of PfRrp6, the recombinant wild-type (WT) PfRrp6 (rPfRrp6) or its variants with two (rPfRrp6-mut2) or four (rPfRrp6-mut4) site mutations at MKIE sites were produced in an Escherichia coli expression system (Fig. 1B). An *in vitro* exoribonuclease assay showed that the four MKIE residues were likely involved in the catalytic activity of PfRrp6, but the mutation of them did not abolish the degradation of the RNA substrate tested (Fig. 1C). More experiments are needed to address this issue.

10.1128/mBio.01110-20.1 FIG S1 Evolution of plasmodial Rrp6 in eukaryotes. (A) Phylogenic tree of the catalytic domain of Rrp6 factor in various eukaryotic organisms. (B) Sequence alignment of the catalytic domain of eukaryotic Rrp6. The putative catalytic residues are highlighted in red. Download FIG S1, TIF file, 2.1 MB. Copyright © 2020 Fan et al. 2020 Fan et al. This content is distributed under the terms of the Creative Commons Attribution 4.0 International license.

Previous studies have identified two RNA exosome-associated (Rrp6 and Dis3) ribonucleases and an exosome-independent (PfRNase II) ribonuclease in P. falciparum by using antibodies generated by animal immunization with synthesized peptides or recombinant proteins (22, 31). Here, we attempted to validate the subcellular interaction between PfRrp6 and the RNA exosome core with PfRNase II as a control by epitope-tagged transfection. The highly conserved core subunit Rrp4 was used as a marker of the exosome core complex. We generated transgenic parasite lines of PfRrp6-Ty1::HA-PfRrp4 and PfRrp6-Ty1::PfRNase II-HA in 3D7 stain by the CRISRP-Cas9 technique (32), and Western blot assay demonstrated that these ribonucleases had been tagged by Ty1 or hemagglutinin (HA), respectively (Fig. 1D and E). Co-immunofluorescence assay (Co-IFA) with antibodies against Ty1 or HA confirmed the direct interaction of PfRrp6 with the exosome complex at the nuclear periphery, whereas PfRNase II appeared to have no significant colocalization with the RNA exosome (Fig. 1F and G), as shown previously (22).

### PfRrp6 knockdown led to a global derepression of heterochromatic genes.

**FIG 2:**
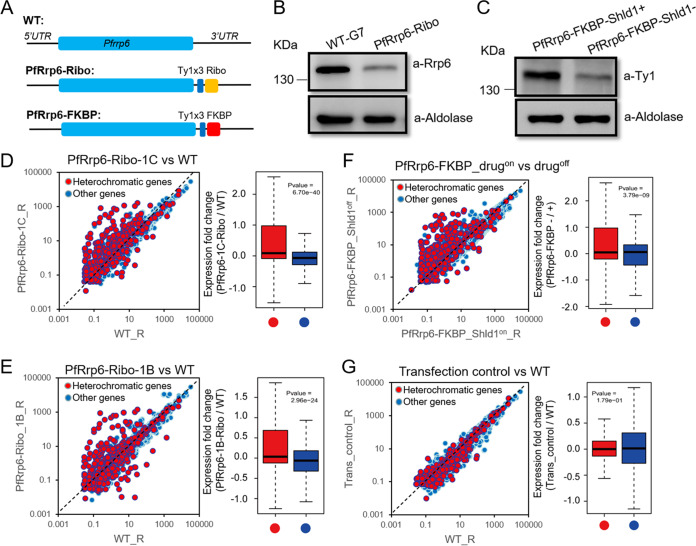
PfRrp6 knockdown derepressed heterochromatic genes. (A) Schematic representation of WT, PfRrp6-Ribo, and PfRrp6-FKBP transgenic parasite lines. (B) Western blot of WT and PfRrp6-Ribo parasites with rabbit antibody against PfRrp6. Aldolase was used as an internal control. (C) Western blot of PfRrp6 protein with antibody against Ty1 for the PfRrp6-FKBP line with or without the drug Shld1. Aldolase was used as an internal control. (D to G) Comparative transcriptome analysis between ring-stage parasites of PfRrp6-Ribo-1C versus WT clone (D), PfRrp6-Ribo-1B versus WT clone (E), PfRrp6-FKBP_drug on versus off (F), and transfection control versus the WT (G). All of the individual HP1-associated genes are indicated by red circles and other genes by blue circles (left). Box plots show expression fold change of HP1-associated genes (red) and other genes (blue) for each comparative strain pair (right). The central line indicates the median, and the whiskers illustrate the interquartile range. *P* values were determined by Wilcox rank test.

In other organisms, Rrp6 is not essential for cell survival (33). However, we failed to obtain *Pfrrp6* knockout parasites after at least three rounds of transfection. Therefore, the conditional gene knockdown strategy was adopted by incorporating the glucosamine-inducible *glmS* ribozyme sequence (34) into the 3′ untranslated region (UTR) of the *Pfrrp6* gene (PfRrp6-Ribo) (Fig. 2A). After transfection, selection, and cloning, we successfully obtained some PfRrp6-Ribo clones. We then carried out RNA-seq analysis of PfRrp6-Ribo-1C and -1B clones at the synchronized ring (R), trophozoite (T), and schizont (S) stages without or with glucosamine treatment for one cycle. The comparative transcriptome analysis showed that no apparent transcriptomic differences were observed upon glucosamine treatment, including that for *Pfrrp6* itself (Fig. S2A and B).

10.1128/mBio.01110-20.2 FIG S2 PfRrp6-Ribo fusion gene triggered knockdown effect at protein level. (A) Global comparison of expression levels for all genes in the PfRrp6-Ribo line with or without drug addition at different IDC stages. (B) Transcriptional level of *rrp6* genes at ring, trophozoite, and schizont stages in PfRrp6-Ribo compared to those of the WT 3D7-G7 clone. (C) Proportions of gene groups differentially expressed in the PfRrp6-ribo-1C clone compared with those of the WT clone at ring stage. The numbers indicate gene counts. (D) Schematic representations of *Pfrrp6-Ribo* and *Pfrrp6-gfpf* constructs. The Ty1-Ribo sequence of PfRrp6-Ribo was replaced by Ty1-GFPf using the CRISPR-Cas9 technique. (E) RT-qPCR assays of *var* genes for ring-stage parasites from PfRrp6-Ribo and PfRrp6-GFP lines, respectively. The relative copy numbers were calculated by the *seryl-tRNA synthetase* gene (PF3D7_0717700). Error bars represent SEM for two biological replicates. Download FIG S2, TIF file, 2.1 MB. Copyright © 2020 Fan et al. 2020 Fan et al. This content is distributed under the terms of the Creative Commons Attribution 4.0 International license.

Strikingly, compared to the WT parent strain 3D7-G7 clone (35), a total of 379 genes were upregulated by 2-fold change (2FC) at ring stage in PfRrp6-Ribo without the addition of glucosamine (Table S1A). Globally, these upregulated genes can be categorized into constitutive-structure ncRNAs and HP1-associated heterochromatic genes (6, 36) (Fig. S2C). Statistical analysis strongly indicated that both PfRrp6-Ribo clones obtained a significant derepression phenotype on heterochromatic genes (Fig. 2D and E), particularly of variant genes like *var*, *rifin*, *stevor*, and *Pfmc-2tm*, and the noncoding *Plasmodium* RNA of unknown function, RUF6 (PlasmoDB) (Fig. S3A and B and S4C). In particular, the multiple expressions of *var* genes may be the consequence of the disruption of mutually exclusive expression patterns or an accelerated switching rate. Analysis on the single-cell level is needed to address this in the future. The 15 members of the RUF6 family previously were described as GC-rich ncRNAs (37–39) that dispersed within the variant gene clusters at chromosomal internal regions (Fig. S4A and B). In addition, for those putative sexual commitment-associated genes identified previously (40–42), only the master regulator, *ap2-g*, was upregulated exclusively throughout the intraerythrocytic development cycle (IDC) (Fig. S3C and S5A). This gene was also regulated by the HP1-dependent heterochromatin environment (6, 36, 43). More importantly, the PfRrp6-Ribo line was capable of producing gametocytes as efficiently as NF54, whereas the parent WT strain was gametocyte deficient because the *ap2-g* gene was almost silent (Fig. S3D). This finding suggests that PfRrp6 knockdown has a fully activated *ap2-g* gene in a subpopulation of parasites, unlike the WT line.

10.1128/mBio.01110-20.3 FIG S3 PfRrp6 knockdown led to a global derepression of heterochromatic genes. (A) Transcriptional profile of *var* gene family of two PfRrp6-ribo clones (1B and 1C), with the WT clone as the control, by RNA-seq analysis. The numbers indicate the expression levels out of the range of the *y* axis. The arrows indicate the individual active *var* products (PfEMP1) detected by Co-IFA in panel B. (B) Comparison of expression levels for three variant gene families, *rifin*, *stevor*, and *Pfmc-2tm*, in PfRrp6-ribo-1C versus the WT clone at trophozoite stages. The data are representative of two biologically independent experiments. (C) *ap2-g* gene expression level in ring (R), trophozoite (T), and schizont (S) parasites of different lines. *P* values were determined by two-tailed Student’s *t* test. ***, *P < *0.001. (D) Dynamic sexual conversion assay of PfRrp6-ribo line with WT 3D7 and NF54 as controls *in vitro*. Error bars represent SEM for three independent assays. (E) The comparative differential transcriptomes of PfRrp6-Ribo and PfRrp6-FKBP clones with regard to WT parasites. Download FIG S3, TIF file, 2.6 MB. Copyright © 2020 Fan et al. 2020 Fan et al. This content is distributed under the terms of the Creative Commons Attribution 4.0 International license.

10.1128/mBio.01110-20.4 FIG S4 Genome-wide distribution of the noncoding *ruf6* genes with regard to variant gene clusters. (A) Localization of all variant gene clusters on individual chromosomes. A total of 33 clusters enriched for *var*, *rifin*, *stevor*, and *Pfmc-2tm* from chromosomes 1 to 13 are shown. Among them, the chromosomal internal clusters containing *ruf6* genes are highlighted in red. (B) All of the chromosomal internal *var* and *ruf6* genes are shown on each corresponding chromosome with regard to the transcriptional orientation of individual genes. Here, only the last five digits of each gene identifier are shown. (C) Transcriptional level of RUF6 ncRNAs in the ring-stage PfRrp6-ribo-1C clone measured by RNA-seq assay. (D) Transcriptional profile of RUF6 ncRNAs in ring-stage RUF6_OE versus the control, measured by RNA-seq assay. Download FIG S4, TIF file, 2.9 MB. Copyright © 2020 Fan et al. 2020 Fan et al. This content is distributed under the terms of the Creative Commons Attribution 4.0 International license.

10.1128/mBio.01110-20.5 FIG S5 PfRrp6 knockdown or RUF6 overexpression activated *ap2-g* gene and rescued gametocytogenesis in WT 3D7-G7 clone. (A and B) Relative expression level of putative gametocytogenesis-associated genes in parasite lines of PfRrp6-Ribo versus WT 3D7-G7 (A) and RUF6_OE versus the control (B), measured by RNA-seq. The *ap2-g* gene is indicated by a red dashed rectangle. Error bars represent SEM for two biological replicates. Download FIG S5, TIF file, 2.3 MB. Copyright © 2020 Fan et al. 2020 Fan et al. This content is distributed under the terms of the Creative Commons Attribution 4.0 International license.

10.1128/mBio.01110-20.8 TABLE S1 Comparative analysis of high-throughput sequencing datasets. (A) Comparative transcriptomes of PfRrp6-Ribo clone versus WT 3D7-G7 clone. (B) Comparative transcriptomes of RUF6_OE clone versus WT 3D7-G7 clone. Download Table S1, XLSX file, 2.2 MB. Copyright © 2020 Fan et al. 2020 Fan et al. This content is distributed under the terms of the Creative Commons Attribution 4.0 International license.

To verify that the generation of the *PfRrp6-Ribo* fusion gene was responsible for this effect, the *glmS* sequence was swapped for a comparably sized sequence from the *gfp* gene (*Pfrrp6-gfp*) from the *PfRrp6-Ribo* locus by CRISPR-Cas9 (Fig. S2D). We then performed reverse transcription-quantitative PCR (RT-qPCR) analysis to profile the transcription of the *var* gene family. As expected, the analysis showed that the mutually exclusive expression of the *var* gene family was rescued in the *Pfrrp6-gfp* clone, i.e., only one member was predominantly expressed (Fig. S2E). Moreover, Western blot assay with antibody against PfRrp6 protein itself revealed that its level was already significantly reduced in PfRrp6-Ribo parasites compared with that of the *Pfrrp6-gfp* line (Fig. 2B). Thus, the fusion of the *glmS* sequence with secondary structure to the 3′ end of the *Pfrrp6* gene might trigger interference with the processing or translation of *Pfrrp6* transcripts. Moreover, we generated another PfRrp6 knockdown line by C-terminal fusion of a destabilization domain, ddFKBP (44), i.e., PfRrp6-FKBP. Western blot assay showed a moderate reduction of PfRrp6 protein level upon treatment with the drug Shld1 (Fig. 2C). Comparative transcriptome analysis confirmed the phenotype observed in the PfRrp6-Ribo line (Fig. 2F and Fig. S3E). In addition, as the heterochromatic genes are mainly composed of variant genes, the analysis needs to exclude the clonal variation of these genes during the manipulation of transfection, drug selection, and cloning. Thus, we generated another transgenic parasite with a gene (PF3D7_0403400, conserved *Plasmodium* protein of unknown function) fused with the *glmS* sequence at the 3′ end as a transfection control of PfRrp6-Ribo. No transcriptomic difference was observed between the transfection control and WT parasites (Fig. 2G).

### PfRrp6 degraded nascent ncRNA RUF6.

**FIG 3:**
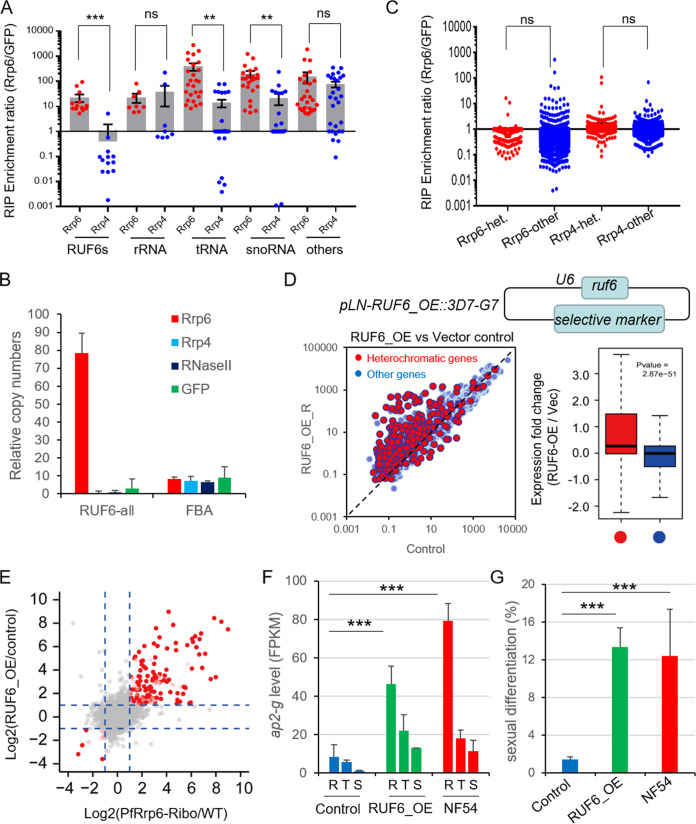
PfRrp6 degraded nascent transcripts of ncRNA RUF6. (A) RIP-seq assays detected specific recognition of RUF6 ncRNAs by PfRrp6, with PfRrp4 and GFP as controls. The synchronized ring-stage transgenic parasites were used. Shown is the statistical analysis of gene enrichment ratios of PfRrp6 or PfRrp4 over the GFP control for different gene groups. The error bars represent medians with 95% confidence intervals. *P* values were determined by two-tailed Student's *t* test. ***, *P < *0.001; **, *P* < 0.01; ns, no significance. (B) RIP-qPCR assay of PfRrp6-Ty1::HA-PfRrp4 (HA antibody for PfRrp4), PfRrp6-Ty1::PfRNase II-HA (Ty1 antibody for PfRrp6, HA antibody for PfRNase II), and GFP-HA-Ty1 control (Ty1 antibody) for RUF6 ncRNAs. The *fructose-bisphosphate aldolase* (FBA) gene (PF3D7_1444800) was used as a control. Error bars represent SEM for three biological replicates. (C) No preferential interaction between heterochromatic genes and PfRrp6 was detected by RIP-seq assays. het., heterochromatic genes; ns, no statistical significance. (D, upper) A schematic representation of a transgenic parasite line of RUF6 overexpression (RUF6_OE). (Bottom) Comparative transcriptome analysis between RUF6_OE and the control at ring stage. (Left) All of the individual HP1-associated genes are indicated by red circles and other genes by blue circles. (Right) Box plots show expression fold change of HP1-associated genes (red) and other genes (blue) from RUF6_OE to vector control lines at ring stage by quantile normalization. The line indicates the median, and whiskers illustrate the interquartile range. *P* values were determined by Wilcox rank test. (E) The comparative differential transcriptomes of PfRrp6-Ribo and RUF6_OE clones with regard to WT parasites. The transcriptome change in levels of PfRrp6-Ribo-1C clone compared to those of the WT clone (*x* axis) and levels of RUF6_OE clone compared to those of the vector control (*y* axis) distinguish cases in which genes that were dysregulated upon PfRrp6-ribo treatment were the same as those of RUF6_OE clones. The dashed lines indicate a fold change cutoff of ≥2 between two samples. Red points show heterochromatin genes that are dysregulated in both the PfRrp6-Ribo clone and RUF6_OE clone. Pink points show other genes that are upregulated or downregulated in both the PfRrp6-Ribo clone and RUF6_OE clone. Gray points show other genes. (F) Transcriptional abundance of the *ap2-g* gene in RUF6_OE, vector control, and NF54 strains at the ring (R), trophozoite (T), or schizont (S). Error bars represent SEM for two biological replicates. *P* values were determined by two-tailed Student's *t* test. ***, *P < *0.001. (G) Gametocytogenesis assay of RUF6_OE line with an *in vitro* negative control and positive control (NF54). The sexual differentiation ratios were calculated by dividing the gametocytemia of mature gametocytes by the parasitemia of all infected RBCs. Error bars represent SEM for three independent assays. *P* values were determined by two-tailed Student's *t* test. ***, *P < *0.001.

The data described above demonstrate that PfRrp6 is associated with heterochromatic gene silencing, but it is unclear whether PfRrp6 degrades them directly. To identify its substrates, we generated a series of epitope-tagging transgenic lines of PfRrp6-Ty1, Ty1-HA-PfRrp4, and GFP-HA-Ty1 (28) and then carried out RIP-seq assay with them at ring and trophozoite stages. Among the enriched genes above a 5-fold enrichment ratio captured by PfRrp6 or PfRrp4, most of them belong to structured ncRNAs, such as rRNAs, tRNAs, and snoRNAs, indicating PfRrp6 together with the RNA exosome core executes evolutionarily conserved functions, as in other eukaryotes (Fig. 3A and Table S2A). Unexpectedly, all the RUF6 ncRNAs were exclusively enriched by PfRrp6, which was confirmed by RIP-qPCR with Rrp4, RNase II, and green fluorescent protein (GFP) as controls (Fig. 3B and Fig. S6A). This result suggests that PfRNase II regulates RUF6 indirectly (22). No significant preferential interaction between PfRrp6 and heterochromatic genes was observed (Fig. 3C), indicating that the transcripts of most heterochromatic genes are not the substrates of PfRrp6. Previous work had shown that *ruf6* genes likely harbored a monoallelic expression mode as *var* genes (39). The RIP-seq data raised the possibility that posttranscriptional degradation by PfRrp6 contributes to the exclusive expression of *ruf6* genes. Indeed, nascent RNA analysis demonstrated the existence of high-level nascent transcripts of individual *ruf6* genes in ring-stage WT parasites (Fig. S6B).

10.1128/mBio.01110-20.6 FIG S6 PfRrp6 recognized RUF6 ncRNAs specifically. (A) RIP-seq signals at individual *ruf6* gene loci for PfRrp6-Ty1, Ty1-HA-PfRrp4, and GFP-HA-Ty1 showing in IGV (Integrative Genomics Viewer). The data are representative of two independent experiments. (B) Comparative qPCR analysis of nascent and steady-state RUF6 ncRNA abundances in ring-stage 3D7-G7 WT parasites. Error bars represent SEM for three biological replicates. Download FIG S6, TIF file, 2.8 MB. Copyright © 2020 Fan et al. 2020 Fan et al. This content is distributed under the terms of the Creative Commons Attribution 4.0 International license.

10.1128/mBio.01110-20.9 TABLE S2 RIP-seq data (A) and oligonucleotide nucleotide sequences used in this study (B). Download Table S2, XLSX file, 1.3 MB. Copyright © 2020 Fan et al. 2020 Fan et al. This content is distributed under the terms of the Creative Commons Attribution 4.0 International license.

As shown in previous studies, the overexpression or suppression of ncRNA RUF6 could activate or silence *var* genes reversibly by unknown mechanisms (38, 39). To expand the potential function of RUF6 ncRNAs in the regulation of all heterochromatic genes, we utilized the same strategy to generate an RUF6-overexpressed clone (RUF6_OE) by episomal transfection. RNA-seq-based transcriptomic analysis of the RUF6_OE clone demonstrated the RUF6 overexpression reproduced the phenotype of the PfRrp6-Ribo line (Fig. 3D, Fig. S4D, and Table S1B), which significantly enhanced *ap2-g*-dependent gametocyte conversion (Fig. 3F and G and Fig. S5B). Finally, the integrated analysis of differential transcriptomes of PfRrp6-Ribo, RUF6_OE, and control lines confirmed that PfRrp6 and RUF6 coregulated the same group of heterochromatic genes (Fig. 3E).

### Accumulated RUF6 ncRNAs triggered local chromatin remodeling *in situ*.

**FIG 4:**
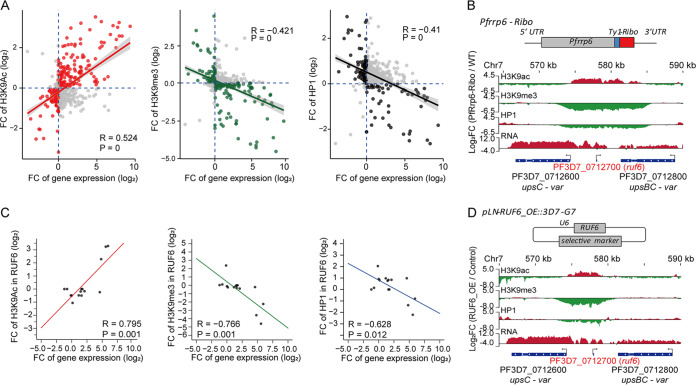
Stabilized RUF6 ncRNAs triggered local chromatin alteration *in situ.* (A) Local chromatin remodeling in the upstream promoter region of heterochromatin genes that are highly expressed in the PfRrp6-Ribo-1C clone (fold change cutoff, ≥2). Scatterplots show the correlation between H3K9ac (left), H3K9me3 (middle), or HP1 (right) level at the 5′UTR of individual heterochromatin gene loci detected by ChIP-seq and the transcription level of corresponding heterochromatin genes detected by RNA-seq. The data are presented as a logarithmic scale of fold changes of PfRrp6-Ribo versus WT clones. (B) Track view of the ratio of the ring-stage PfRrp6-ribo parasites to the WT for H3K9ac, H3K9me3, and HP1 and expression levels in the central chromosomal *var* gene loci. The transcriptional orientations of these genes are labeled by arrows. (C) Correlation between H3K9ac, H3K9me3, or HP1 level at individual *ruf6* gene loci and transcriptional level of the corresponding RUF6 ncRNA. The data are presented by logarithmic scale of fold changes of PfRrp6-ribo-1C versus the WT clone. *P* values were calculated by cor.test in R. (D) Track view of the ratio of the ring-stage RUF6_OE parasites to the control line for H3K9ac, H3K9me3, and HP1 and expression levels in the central chromosomal *var* gene loci. The transcriptional orientations of these genes are labeled by arrows.

In P. falciparum, histone modifications control the transcriptional state of variant genes, e.g., the binding of PfHP1 to H3K9me3 established repressive heterochromatin, while H3K9ac is associated with transcriptionally permissive euchromatin (6, 12). The alteration in the local chromatin microenvironment induced variant expression of *var* genes (15). To explore whether the disruption of variant gene expression in the PfRrp6-Ribo line is associated with chromatin remodeling, a series of chromatin immunoprecipitation and high-throughput sequencing (ChIP-seq) assays for HP1, H3K9me3, and H3K9ac were performed in ring-stage PfRrp6-Ribo and WT clones with antibodies against these histone markers. The genome-wide dynamics of histone modifications showed that the H3K9me3 level was significantly reduced in most heterochromatin regions of the PfRrp6-Ribo line, whereas H3K9ac levels increased accordingly (Fig. S7A). This finding suggested an opposite regulatory function of PfRrp6 (repression) and RUF6 (activation) for heterochromatic genes. Moreover, we observed a positive correlation between heterochromatin gene activation and local chromatin remodeling from the heterochromatic (H3K9me3 or HP1) to euchromatic (H3K9ac) microenvironment (Fig. 4A).

10.1128/mBio.01110-20.7 FIG S7 Stabilized RUF6 stimulated local chromatin remodeling at promoters. (A) Track view of H3K9ac and H3K9me3 signals in each chromosome of the PfRrp6-Ribo-1C clone normalized to the WT control. Red, H3K9ac. Blue, H3K9me3. (B) Composite distribution of H3K9ac, H3K9me3, and HP1 relative to TSS of highly activated *var* genes in the PfRrp6-Ribo-1C line and the WT control. (C) RUF6 ncRNAs triggered the local chromatin remodeling in the upstream promoter region of *var* genes. Scatter plots show the correlation between H3K9ac, H3K9me3, and HP1 levels at different regions (5′UTR, gene body, and 3′UTR) of individual *var* gene loci detected by ChIP-seq and the transcription level of corresponding *var* genes detected by RNA-seq, respectively. The data are presented on a logarithmic scale of fold changes of PfRrp6-Ribo-1C versus WT clones. Download FIG S7, TIF file, 2.9 MB. Copyright © 2020 Fan et al. 2020 Fan et al. This content is distributed under the terms of the Creative Commons Attribution 4.0 International license.

We also found that local chromatin remodeling mainly occurred at the upstream region of heterochromatic genes in either the PfRrp6-Ribo or RUF6_OE line. As an example, statistical analysis revealed a positive correlation between transcriptional level and H3K9ac modification at the 5′UTR of *var* genes and a negative correlation for H3K9me3 or HP1 markers (Fig. S7B and C). Intriguingly, the local chromatin alteration occurred in the *ruf6* and chromosomal central *var* gene cluster, suggesting that the accumulated RUF6 upon PfRrp6 knockdown triggered chromatin remodeling, thereby activating its adjacent *var* gene in a head-to-head orientation (Fig. 4B). A consistent pattern was also observed for ncRNA RUF6 levels and the local chromatin modifications at *ruf6* loci (Fig. 4C). This phenomenon was also observed in the RUF6_OE line (Fig. 4D), which confirmed the previous finding that the abundance of RUF6 ncRNAs was critical for the transcriptional activity of *var* genes.

### ChIRP-seq revealed direct interaction between ncRNA RUF6 and heterochromatin regions.

**FIG 5:**
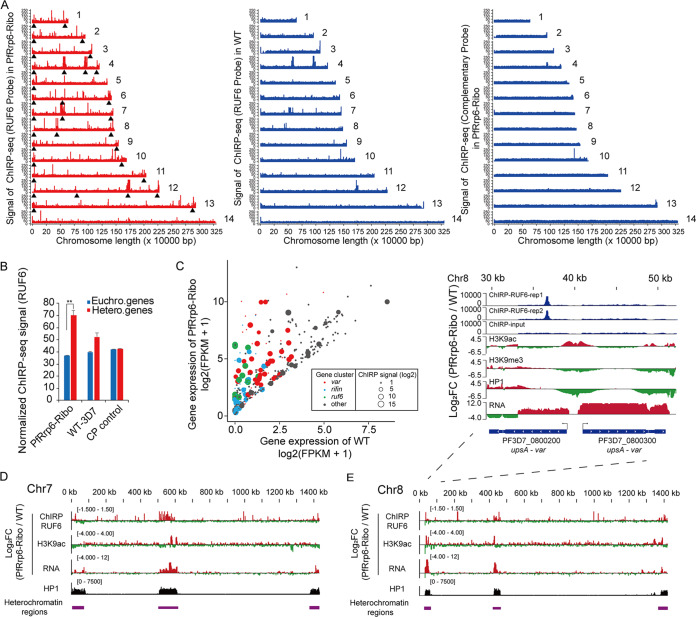
ncRNA RUF6 interacted with heterochromatic gene loci directly. (A) Overview of genome-wide ChIRP-seq signals of RUF6 ncRNAs for PfRrp6-ribo (left) and WT 3D7-G7 (middle) clones with a complementary probe control (right). The typical heterochromatin domains enriched by variant gene families are indicated by black triangles. (B) Bar graph illustrates the normalized frequencies of ncRNA-genomic DNA interactions for RUF6 ncRNAs with the euchromatic or heterochromatic gene loci. ChIRP-seq data of PfRrp6-ribo-1C, WT, and complementary probe (CP) control were used. *P* values were determined by two-tailed Student's *t* test. **, *P* < 0.01. (C) The scatter diagram displays the RUF6 ChIRP signal enrichment on different clusters of genes that were upregulated in PfRrp6-ribo compared to levels of the WT in the ring stage. The size of points is based on ChIRP signal intensity. The color of points is based on different clusters. (D and E) Track view of RUF6 binding, H3K9ac enrichment, and gene abundance changes of two representative chromosomes (Chr.7 [D] and Chr.8 [E]) for PfRrp6-Ribo-1C versus the WT. The heterochromatin regions on each chromosome are marked with purple rectangles with regard to the distribution of HP1 ChIP signals. Detailed information on the RUF6 signals, chromatin alteration, and gene expression changes for a representative region at the Chr.8 subtelomere are shown at the top of panel E.

In addition to the central *var* genes located in *ruf6-var* gene clusters, other heterochromatic genes in central or subtelomeric regions were also upregulated in the PfRrp6-Ribo line. To gain insight into the regulatory role of the *trans*-acting RUF6 ncRNAs on heterochromatic genes, we adopted a chromatin isolation by RNA purification technique (ChIRP-seq) (45, 46) to identify those interacting targets of RUF6 in the PfRrp6-Ribo line with WT parasites or complementary probes as controls. Strikingly, ChIRP-seq data showed that the accumulated RUF6 ncRNAs produced in chromosomal internal regions had interacted with distal subtelomeric heterochromatic regions directly, whereas the majority stayed in the original loci in the WT parasites. No significant enrichment signal was observed with the complementary tiling probes (Fig. 5A). Genome-wide statistical analysis of RUF6 ChIRP signals confirmed the preferential binding of RUF6 on heterochromatic genes, most of which belong to variant gene families (Fig. 5B and C). Integrative analysis of ChIPR-seq, ChIP-seq, and RNA-seq suggests a regulation cascade of ncRNA RUF6 accumulation, interaction between RUF6 and the distal heterochromatin region, chromatin alteration, and transcriptional activation for heterochromatic genes (Fig. 5D and E).

Recently, the adoption of a Hi-C-seq technique for malaria parasites revealed the highly frequent interaction of inter- or intrachromosomal heterochromatin regions (47, 48). Given this fact, we speculate that the higher-order chromatin structures provide a scaffold for the occupancy of the stabilized RUF6 ncRNAs in distal heterochromatic regions and then may activate those subtelomeric variant genes or the *ap2-g* gene via antagonizing the HP1-dependent heterochromatin microenvironment.

## DISCUSSION

The RNA exosome complex is highly conserved in composition, structure, and function in various eukaryotes (49). In the eukaryotic parasites, such as P. falciparum, we previously discovered a homologous complex, although a few subunits were absent from the exosome core (28). Here, we further reveal that although the catalytic residues DEDD are replaced by MKIE, PfRrp6 still harbors a structure and enzymatic activity similar to those of yeast or human Rrp6. It also executes the conserved function of structured RNA processing *in vivo*, e.g., tRNAs and rRNAs. Unexpectedly, PfRrp6 exhibits unique characteristics in the human malaria parasite, i.e., it degrades the nascent transcripts of ncRNA RUF6 to maintain the mutually exclusive expression of this noncoding gene family. Given the facts that RUF6 is a critical regulator in the counting mechanism of the *var* gene family (38, 39) and PfRrp6 is required to secure the proper expression of ncRNA RUF6, plasmodial Rrp6 is involved in the heterochromatic gene silencing indirectly. Therefore, such a mechanism confers this exosome-associated Rrp6 with a critical role in cell fate decisions by the regulation of heterochromatic gene expression. In addition, no significant interaction of the exosome core (Rrp4) with nascent RUF6 was found (Fig. 3A and B), suggesting an exosome-independent function of PfRrp6 in regulating RUF6 levels. More structural details of PfRrp6-RUF6 interaction should be investigated in future work.

**FIG 6:**
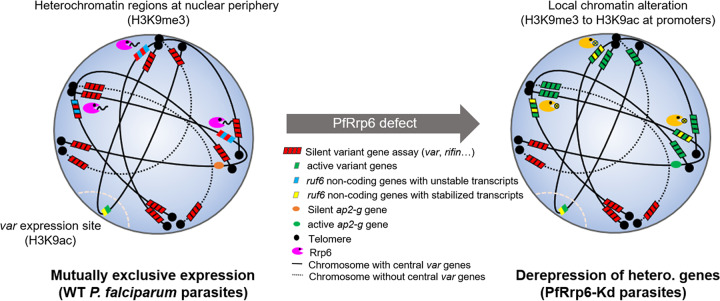
Putative model of PfRrp6 in regulating heterochromatic gene silencing. In the wild-type parasites (left), PfRrp6 degrades the nascent unstable RUF6 ncRNAs, which secures the mutually exclusive expression of those variant gene families and low transcription abundance of the *ap2-g* gene. When PfRrp6 was downregulated (right), the stabilized and accumulated RUF6 ncRNAs would trigger local chromatin remodeling *in situ* and then derepressed distal heterochromatic genes, including subtelomeric variant genes and the *ap2-g* gene, probably via the higher-order chromosome organization as the scaffold.

In model organisms such as yeast, *Drosophila*, or Caenorhabditis elegans, constitutive heterochromatin traditionally has been acknowledged as a static chromatin structure in the maintenance of gene silencing (5). Recent studies have shown its dynamics in response to various stimuli accompanied by the fluctuation of HP1 and H3K9me3 levels (14), but the mechanisms of heterochromatin surveillance are poorly understood. Unexpectedly, in the malaria parasites, we, for the first time, identified the conserved Rrp6 as a heterochromatin surveillant via degradation of nascent RUF6 ncRNAs, which is vital for immune evasion and transmission of this parasite. The higher-order heterochromatin structure in the nucleus governs gene silencing, and PfRrp6 constrains RUF6 ncRNA levels to maintain the heterochromatin state (Fig. 6). Therefore, the conserved Rrp6 has evolved new biological functions in malaria parasites. The first task of PfRrp6 is to control the strict mutually exclusive expression of RUF6 ncRNAs, as observed in PfRNase II-mediated silencing of *upsA-var* genes (22). Next, due to the critical role of RUF6 ncRNAs in the activation of heterochromatic genes, PfRrp6 harbors a second role in regulating heterochromatic gene silencing. A previous report had observed that the overexpression of different RUF6 ncRNAs was able to activate a fixed set of multiple *var* genes, including some subtelomeric *var* genes; thus, they proposed that the *var* gene family has a hard-wired activation program (38). Our finding may interpret that observation as the regulatory role of *ruf6-var* pairs, but it also suggests that such artificial conditions established by overexpression do not reflect the nature of *var* gene expression. For instance, a *ruf6*-independent regulation pathway for those *upsB*-*var* genes may exist. A recent study has demonstrated that once a non-*ruf6*-associated *var* gene was predominantly expressed, the entire *ruf6* gene family had been silenced in parasite clones (39). Even though we do not know how the two groups of *var* genes are coordinately expressed, *ruf6-var* pairs likely contribute to driving expression switching during the clonal variation of the *var* gene family.

Previously, we found that the RNA exosome-independent RNase PfRNase II regulates the mutually exclusive expression of the *var* gene family via the degradation of nascent *var* transcripts *in situ*. Strikingly, some heterochromatic genes, including the ncRNA RUF6, were also upregulated in the PfRNase II-deficient line. This finding raised the possibility that PfRrp6 is coordinated with PfRNase II for the regulation of heterochromatic genes. However, our data showed that PfRNase II did not degrade ncRNA RUF6 directly (Fig. 3B). Together with the fact that no significant subcellular interaction between the two ribonucleases was observed (Fig. 1F and G), we speculate that the main task of PfRNase II is silencing the *upsA*-subtype *var* genes, but the activated *var* promoters may influence the expression, since they are colocalized in the nuclear periphery through chromosome organization. The upregulated RUF6 then triggered the downstream phenotype, similar to that of the PfRrp6-Ribo line. Therefore, although it is unclear whether the two pathways are coordinated, both of the ribonucleases are involved in the posttranscriptional regulation of heterochromatic genes.

The local chromatin structure of the 5′UTR had been shown to regulate the transcriptional activity of *var* genes. Even for the typical heterochromatin marker H3K9me3, significant differences were observed only at the 5′ flanking region of a given *var* gene in distinct transcriptional states (11). Consistent with these observations, our data demonstrate that local chromatin remodeling between heterochromatic and euchromatic modifications at the 5′UTR is sufficient to activate the downstream *var* gene. Consequently, the expression switching of *ruf6* genes can stimulate clonal variation of *var* genes. Here, two questions were raised by this finding. First, which factor is regulating the expression switching of *ruf6* genes? It may virtually determine the selection of individual *var* genes for predominant expression. Second, which chromatin remodeling factor has been recruited by the ncRNA RUF6? We have tried to identify this factor *in vitro* by RNA pulldown and liquid chromatography-tandem mass spectrometry (LC-MS/MS), but no proteins of interests were found. An *in vivo* analysis, such as ChIRP followed by LC/MS-MS, may be used in this case.

In conclusion, the present study unveils a unique and critical function of the evolutionarily conserved Rrp6 in human malaria parasites. The underlying mechanism will open a new avenue for understanding the multiple layers of the complex gene expression program in this pathogen. In addition, our findings expand our knowledge of the biological roles of the evolutionarily conserved Rrp6 factor in regulating the fate of eukaryotic cells, which will provide an important target of antimalarial drugs or vaccine development.

## MATERIALS AND METHODS

### Plasmid construction for transfection.

To generate PfRrp6-Ribo, PfRrp6-FKBP, PfRrp6-Ty1, PfRrp6-gfp, Ty1-HA-PfRrp4, PfRNase II-HA-Ty1, and PfRrp6-Ty1::HA-PfRrp4 transgenic lines, we modified the plasmid *pL6-gfp* by replacing the *gfp* box with an ∼1-kb homologue sequence flanking the N or C terminus of the target genes that contained three copies of the epitope (HA or Ty1) or a single fragment of the *gfp* gene and inserting a guide RNA sequence specific to the *Pfrrp6* gene (PF3D7_1449700), *Pfrrp4* gene (PF3D7_0410400), or *Pfrnase II* gene (PF3D7_0906000) by the In-Fusion PCR cloning system (see Table S2B in the supplemental material). The resulting plasmids were *pL6-Pfrrp6-Ty1-Ribo*, *pL6-Pfrrp6-Ty1-FKBP*, *pL6-Pfrrp6-Ty1*, *pL6-Pfrrp6-gfp*, *pL6-Ty1-HA-Pfrrp4*, *pL6-HA-Pfrrp4*, and *pL6-Pfrnase II-HA-Ty1*. The plasmid pUF1-Cas9-infusion, carrying the Cas9 expression cassette, was modified by replacing *ydhodh* with the *hdhfr* gene (32).

### Parasite culture and transfection.

P. falciparum strain 3D7-G7 was maintained in culture *in vitro* and synchronized as described previously (35). For transfection, synchronized ring-stage parasites at ∼5% parasitemia were transfected with ∼100 μg of plasmid single guide RNA and Cas9 by electroporation as described previously (32). Transfected parasites were selected by blasticidin S deaminase (BSD) and WR99210. After approximately 4 weeks, the established cultures were subcloned by limiting-dilution cloning, and the integration events were examined by PCR followed by DNA sequencing.

### Production of recombinant exoribonucleases.

The DNA fragment of the exoribonuclease catalytic domain of PfRrp6 (residues 531 to 740) was amplified from cDNA (WT 3D7-G7) as the template with the primers shown in Table S2B. The resulting PCR product was cloned into the vector *pGEX-4T-1* by BamHI and EcoRI sites, followed by transformation into Escherichia coli BL21(DE3). The inducible expression of recombinant protein and affinity purification were performed in accordance with the manufacturer’s recommendations (GE Healthcare). The dead mutation of PfRrp6 was generated by PCR mutagenesis.

### *In vitro* exoribonuclease assay.

The exoribonuclease reactions were performed as described previously (22). In brief, the 17-mer oligoribonucleotides (5′-CCCCACCACCAUCACUU-3′) were labeled at their 5′ ends with biotin (Invitrogen). The assays were performed in 10-μl reaction mixtures containing the recombinant PfRrp6 protein and substrate at concentrations of 6 and 40 μM, respectively. Reaction mixtures were incubated at 37°C for the indicated durations, and they were stopped by adding loading buffer containing 30 mM EDTA (TaKaRa). Reaction products were resolved in 15% (wt/vol) polyacrylamide gel with 5 M urea and then transferred to the nylon membrane and detected with a chemiluminescent nucleic acid detection module (Thermo).

### Immunofluorescence.

Immunofluorescence was performed as described previously (35). Infected red blood cells were lysed with 0.15% saponin, and the released parasites were fixed in suspension with 4% paraformaldehyde for 20 min on ice. Parasites then were deposited on microscope slides and subjected to IFA. The antibody dilution for mouse anti-Ty1 was 1:300 and for rabbit anti-HA was 1:200. Alexa-Fluor 488 (or 568)-conjugated anti-mouse or anti-rabbit secondary antibody was diluted 1:2,000. Images were captured by using a Nikon A1R microscope at ×100 magnification. NIS Elements software (Nikon) was used for acquisition and Image J (http://rsbweb.nih.gov/ij/) for composition.

### Nascent RNA analysis.

The capture of nascent RNA was performed as described previously (22). The biotin-labeled nascent RNA was purified by Dynabeads MyOne streptavidin T1 magnetic beads. The purified nascent RNA was reverse transcribed into cDNA with random primers or specific primers for mRNA. The abundance of nascent RNA or steady-state RNA was measured by RT-qPCR.

### Gametocytogenesis assay.

Gametocyte cultures were established as described previously (50). Briefly, the starter culture for gametocytes was 4% hematocrit (fresh human blood not more than a week old), and parasites were grown to ∼1% parasitemia in 6-well plates. Five milliliters of complete medium with 0.5% AlbuMax II was changed every day without disturbing the RBC layer at the bottom of the well. The growth of the parasites was monitored by making smears every day until day 14. Slides were counted and gametocyte parasitemia was calculated. Each experiment had three biological replicates.

### High-throughput sequencing-associated analysis.

The technical details of chromatin immunoprecipitation and high-throughput sequencing (ChIP-seq), chromatin isolation by RNA purification (ChIRP-seq), RNA immunoprecipitation and high-throughput sequencing (RIP-seq), RNA-seq, and related bioinformatics analyses are available in the supplemental material.

### Data availability.

All data are available in the main text or the supplemental material. The high-throughput sequencing data of this study have been deposited in the Gene Expression Omnibus (GEO) database under accession number GSE133241.

10.1128/mBio.01110-20.10 TEXT S1 High-throughput sequencing-associated analysis. The technical details of chromatin immunoprecipitation and high-throughput sequencing (ChIP-seq), chromatin isolation by RNA purification (ChIRP-seq), RNA immunoprecipitation and high-throughput sequencing (RIP-seq), RNA-seq, and related bioinformatics analysis are available in the supplemental methods. Download Text S1, DOCX file, 0.02 MB. Copyright © 2020 Fan et al. 2020 Fan et al. This content is distributed under the terms of the Creative Commons Attribution 4.0 International license.
