# Supplementary material for: Rrp6 Regulates Heterochromatic Gene Silencing via ncRNA RUF6 Decay in Malaria Parasites
Source: mBio. 2020 Jun 2;11(3):e01110-20. doi: 10.1128/mBio.01110-20 (PMC7267889; doi:10.1128/mBio.01110-20)
Supplement: TEXT S1 [file mBio.01110-20-s0001.docx]

**Supplementary Materials and Methods**

**ChIP-seq**. Synchronized PfRrp6-Ribo, RUF6_OE, and WT parasites at different stages were used for ChIP assay. The antibodies against H3K9me3 (Upstate#07-442), H3K9ac (Upstate# 07-352), PfHP1 (51) were used in this study. Parasite culture (1x10^9^ rings) was harvested and cross-linked immediately with 1% paraformaldehyde with rotation for 10 min at 37 ℃, and quenched with 0.125 M glycine for 5 min on ice. The fixed culture was washed twice with cold 1 x PBS, and the parasites were released from iRBC with 0.15% saponin for 15 min on ice. After washed with cold 1 x PBS, the nuclei were isolated by incubation with 2 ml of cold Lysis Buffer (10 mM HEPES pH 7.9, 10 mM KCl, 0.1 mM EDTA pH8.0, 0.1mM EGTA pH8.0, 1mM DTT, 0.25% N-P40, 1 x protease inhibitors cocktail) for 30 min on ice, and followed by dounce homogenization for 200 strokes. After centrifugation at 13,000 g for 10 min at 4 ℃, the nuclei were resuspended in 150 μl of SDS Lysis Buffer (1% SDS, 10 mM EDTA, 50 mM Tris-HCl pH8.0) for sonication. Chromatin was sheared into 200-500 bp fragments with Bioruptor (Bioruptor^TM^ UCD-200) of highest power for 4 x 4 min at 30 sec intervals. The resulting chromatin was diluted 1:10 in ChIP dilution buffer (0.01% SDS, 1.1% Triton X-100, 1.2 mM EDTA, 16.7 mM Tris-HCl pH8.0, 150 mM NaCl, 1 x protease inhibitors cocktail). Pre-clear the chromatin solution with 50 μl per ml of salmon sperm DNA/protein A/G agarose slurry for 2 h at 4 ℃ with agitation. After centrifugation, per 250 μl of chromatin supernatant was incubated with 3 μg of Rabbit anti-H3K9me3 antibody (Abcam, ab8898), 3 μg of Rabbit anti-H3K9Ac antibody (Millipore, 07-352) and 0.5 μg of Rabbit anti-PfHP1 for each IP at 4 ℃ overnight and rabbit IgG (Sigma) as control. To collect immune complexes, the sample was incubated with 50 μl of Salmon sperm DNA/protein A/G slurry for 2 h at 4 ℃ with agitation. After extensive washes, immunoprecipitated chromatin was eluted with ChIP Elution Buffer (1% SDS, 0.1 M NaHCO_3_), and reverse-crosslinked at 65 ℃ for 16 h in the presence of 200 mM NaCl, then added RNase A at 37 ℃ for 30 min and incubated with the proteinase K at 45 ℃ for 2h. DNA was purified by Phenol/chloroform/isoamylic alcohol extraction for ChIP-seq.

To prepare sequencing libraries, the purified DNA were end repaired, extended with 3′ A-overhangs and ligated to barcoded NextFlex adapters (Bio Scientific). Libraries were amplified using KAPA HiFi HotStart ready mix (KAPA Biosystems) and the following PCR program: 98 ℃ for 3 min followed by 14 cycles of 98 ℃ for 20 sec, 65 ℃ for 30 s; 68 ℃ for 30 s; finally extended 68 ℃ for 5 min. Amplified libraries were size-selected for 300-600 bp using 2% agarose gels. The final libraries were sequenced on an Illumina HiSeq Xten systerm to generate 150 bp pair-end reads.

**ChIRP-seq.** The ChIRP assay was performed as described previously (46) with some modifications. 1x10^9^ synchronized ring-stage (15 ± 5 h post invasion) parasites were cross-linked with 1% paraformaldehyde with rotation for 10 min at 37 ℃, and quenched with 0.125 M glycine for 5 min on ice. After three washes with cold 1 x PBS, the parasite pellets were lysed with the swelling buffer (0.1 M Tris pH 7.0, 10 mM KOAc, 15 mM MgOAc, 1% NP-40, 1 mM DTT, 1 x protease inhibitors, and 0.2 U/μl RNase inhibitor) at 4 ℃ for 10 min with rotation. After centrifugation at 13,000 g for 10 min at 4 ℃, the parasites were resuspended in 150 μl of SDS Lysis Buffer (1% SDS, 10 mM EDTA, 50 mM Tris-HCl pH 8.0) for sonication. Chromatin was sheared into 100-500 bp fragments with Bioruptor (Bioruptor^TM^ UCD-200) of highest power for 4 x 4 min at 30 sec intervals. Chromatin was diluted in 2 x volumes of hybridization buffer (500 mM NaCl, 1% SDS, 100mM Tris 7.0, 10 mM EDTA, 15% Formamide, add 1 mM DTT, 1 x protease inhibitors and 0.2 U/μl RNase inhibitor). 100 pmol probes were added to each reaction, which was mixed by end- to-end rotation at 37 ℃ for 4 h. Dynabeads® MyOne™ Streptavidin C1 beads (Invitrogen) were washed three times in SDS lysis buffer, and then blocked with 500 ng/μl yeast total RNA and 1 mg/ml BSA for 1 h at 37 ℃, and washed three times again in SDS lysis buffer before resuspended in its original volume. 100 μl of washed/blocked C1 beads were added per 100 pmol of probes, and the whole reaction was mixed for another 45 min at 37 ℃. Beads:biotin-probes:RNA:chromatin products were captured by magnets (Invitrogen) and washed five times wash buffer (2 x SSC, 0.5% SDS, add 1 mM DTT and 1 x protease inhibitors, fresh). After last wash buffer was removed carefully with P-10 pipette, beads were resuspended in 3 x original volumes of DNA elution buffer (50 mM NaHCO_3_, 1% SDS, 200 mM NaCl), and DNA was eluted with 100 μg/ml RNase A and 0.1 U/μl RNase H at 37 ℃ with end-to-end rotation. Then chromatin was reverse-crosslinked with 0.2 U/μl protease K at 65 ℃ for overnight. DNA was then extracted with equal volume of phenol:chloroform:isoamyl. Eluted DNA was subject to high-throughput sequencing. ChIRP-seq libraries were constructed following the protocol of ChIP-seq (see above) and sequenced using an Illumina Novaseq systerm to generate 150 bp pair-end reads.

**RIP-seq.** RIP assays were performed as described previously (52) with some modifications. Briefly, an equivalent of 5 x 10^9^ synchronized parasites were collected by saponin lysis. The resulting parasite pellet was lysed under non-denaturing conditions (50 mM Tris-Cl pH7.4, 150 mM NaCl, 1 mM EDTA, 1 mM EGTA, 1% Triton X-100/NP-40, 1 x Proteinase inhibitors, 0.2 U/μl RNase inhibitor) for 2 h at 4 ℃ with rotation, then the supernatant was collected by centrifugation at 13,000 g for 15 min at 4 ℃, and subjected to immunoprecipitation analysis with 10 μg mouse anti-Ty1 for 3 h at 4 ℃, then incubated with the protein G magnetic beads (Thermo) at 4 ℃ for overnight. After washing the beads twice with IPP500 (500 mM NaCl, 10 mM Tris-Cl pH 8.0, 0.05% NP-40, 1 x Proteinase inhibitor, 0.2 U/μl RNase inhibitor) and once with 1 x PBS, bounded RNA was eluted by TRIzol reagent for 10 min with rotation at 4 ℃, then RNA was extracted by phenol-chloroform method and treated with DNase I for 10 min at room temperature. The RNA was directly used to prepare strand-specific RNA-seq libraries (see below) without poly (A) enrichment. A minimum two biological replicates were sequenced for each experiment and control.

**RNA-seq.** The RNA isolation, mRNA enrichment and library construction were performed as described using 15 cycles of library amplification (53). In brief, synchronized parasites were harvested, and total RNAs were isolated with a Direct-zol RNA Kit (Zymo Research). mRNA was enriched by poly(A) selection with the KAPA mRNA Capture Beads (KAPA), and fragmented to about 300–400 nucleotides (nt) in length, then all subsequent steps were performed in accordance with an KAPA Stranded mRNA-Seq Kit Illumina platform and sequenced on an Illumina HiSeq Xten systerm.

**Strand-specific RNA-seq data analysis.** Low-quality and adaptor sequences were trimmed from the reads using cutadapt (v1.16) (54) with parameters: -a AGATCGGAAGAGC -AAGATCGGAAGAGC --trim-n -m 75 -q 20,20. Then, the reads were mapped to the *Plasmodium falciparum* 3D7 genome (Pf 3D7 v32, obtained from PlasmoDB) using Hisat2 strand-specific mode (v2.1.0) (55) with parameters: --rna-strandness RF --dta --no-discordant --no-mixed --no-unal. The Samtools (v1.9) (56) was used to transfer the mapping results from sam format to position sorted bam format. After that, Mapped reads were subsequently assembled into transcripts guided by the PlasmoDB gff annotation files (Pf 3D7 v32) using featureCounts (v1.6.1) (57) with parameters: -M -p -B -C for all; -s 2 for sense transcripts; -s 1 for antisense transcripts. Finally, the expression level of each gene was quantified and normalized as FPKM (fragments per kilobase of transcript sequence per million read pairs mapped) based on featureCounts raw outcome in R. Both sense and antisense read counts were merged for library normalization. Additionally, the bam files were converted to bigwig files using bamCoverage from the deeptools suite (58) (v3.1.3) with parameters: --normalizeUsing RPKM --binSize 10 --smoothLength 30 -ignore Pf3D7_API_v3 Pf_M76611. The bam files were splited into sense and antisense using featureCounts with parameters: –R bam; -s 2 for sense transcripts; -s 1 for antisense transcripts. The Integrative Genomics Viewer (IGV) (59) was used to show the signal of gene expression in certain genomic region in a track view.

**RIP-seq data analysis (strand-specific).** Sequencing reads were trimmed similarly as in the above RNA-seq data analysis, with one modified parameter: –m 50. The read mapping, signal quantification, and file format conversion were done similarly as in the above RNA-seq data analysis. The traditional RNA-seq was generated for each sample as input control.

**ChIP-seq data analysis (Histone modifications (HM) and HP1).** Low-quality and the adaptor sequences were trimmed from the reads using cutadapt (v1.16) with parameters: -a AGATCGGAAGAGC -A AGATCGGAAGAGC --trim-n -m 50 -q 20,20. Then, the reads were mapped to the *Plasmodium falciparum* 3D7 genome (PlasmoDB v32) using Bowtie2 (v2.3.4.3) (60) with parameters: -N 0 --no-discordant --no-mixed --no-unal. The Samtools (v1.9) was used to transfer the mapping results from sam format to position sorted bam format. Next, the duplicated reads were removed by markdup from sambamba (v0.6.8) (61). Then, the bam files were converted to bigwig files using bamCoverage from the deeptools suite (v3.1.3) with parameters: --normalizeUsing RPKM --binSize 10 --smoothLength 30 -ignore Pf3D7_API_v3 Pf_M76611. The bigwig files from ChIP sample were normalized to input sample by bigwigCompare from the deeptools suite (v3.1.3) with parameters: –operation log2 --binSize 10 --pseudocount 1. The Integrative Genomics Viewer (IGV) was used to show the signal of histone modification in certain genomic region in a track view.

**The global HM signals in each chromosome.** Each chromosome was split into a number of bins using makewindows from bedtools (62) (v2.27.1) with parameter -w 100. The raw counts of HM ChIP-seq reads in each bin were calculated by multiBamSummary BED mode with the parameter centerReads from the deeptools suite (v3.1.3). The read counts were further normalized as FPKM and transformed as log_2_(FPKM+1). Finally, the input signals were subtracted from the HM signals. The resultant difference was plotted by the geom_line from ggplot2 in R.

**The distribution of ChIP signals around genes.** The distribution of ChIP signals around genes were generated by computeMatrix and plotProfile from the deeptools suite (v3.1.3). To do so, all *var* genes were scaled to the same length of 4000 bp. 2500 bp upstream of ATG and 1500 bp downstream of the stop codon were also included. The input data files for computeMatrix and plotProfile were the bigwig files of the ChIP signals normalized by the input sample (see the above “ChIP-seq data analysis”).

**Correlation between ChIP signals and gene expression levels.** For *var* genes, we defined the upstream 2000 bp from ATG as 5’UTR, downstream 1500 bp from stop codon as 3’UTR, the region from ATG to stop codon as gene body. The raw counts of HM ChIP-seq reads in the three regions were calculated by multiBamSummary BED mode with the parameter centerReads from the deeptools suite (v3.1.3), respectively. The read counts were further normalized as FPKM. The Pearson correlations between the normalized HM signals in these three regions and the gene expression levels (see the above RNA-seq data analysis) were calculated, respectively. The statistical significance was evaluated by cor.test in R. The scatter plot was drawn by geom_point from ggplot2 in R. The geom_smooth from ggplot2 in R was used to fit regression. As for RUF6 genes, the region from upstream 500 bp of ATG to downstream 500 bp of stop codon was used. The normalized HM signals in this region for each RUF6 gene were calculated and normalized as above regions of *var* genes. The Pearson correlations between the normalized HM signals in this region and the gene expression levels were calculated and presented as above regions of heterochromatin genes.

**ChIRP-seq data analysis.** Sequencing reads were trimmed and then mapped to the *Plasmodium falciparum* 3D7 genome (Pf 3D7 v32) similarly as in the above ChIP-seq data analysis. Genome-wide ChIRP-seq signals were calculated using a 10-bp window and normalized to the uniquely mapped fragments using ‘bamCoverage’ from deepTools (v3.1.3). ChIRP peaks were predicted by MACS2 (v2.1.1) (63) with Q-value < 0.01 and the parameter: -c.

**Validate reproducibility of ChIRP-seq data.** Pearson correlation was used to calculate the reproducibility score between the libraries of two replicates to validate the reproducibility of the ChIRP data. We first scanned the whole genome with 1-kb windows and calculated ChIRP signal using deeptools suite multiBigwigSummary (v3.1.3). Then we use pearson correlation to calculate the value and ‘pheatmap’ to show them.

**The global ChIRP signals in each chromosome.** The analysis of global ChIRP signals in each chromosome similarly as in the above ChIP-seq data analysis. In brief, each chromosome was split into a number of bins using makewindows from bedtools (v2.27.1) with parameter -w 1000. The raw counts of ChIRP-seq reads in each bin were calculated by multiBamSummary BED mode from the deeptools suite (v3.1.3). The read counts were further normalized as FPKM and transformed as log_2_(FPKM+1). The values were plotted by the geom_line from ggplot2 in R. The experiment of complementary probe was used as negative control.

**Supplementary References**

51. Zanghi G, Vembar SS, Baumgarten S, Ding S, Guizetti J, Bryant JM, Mattei D, Jensen ATR, Renia L, Goh YS, Sauerwein R, Hermsen CC, Franetich JF, Bordessoulles M, Silvie O, Soulard V, Scatton O, Chen P, Mecheri S, Mazier D, Scherf A. 2018. A Specific PfEMP1 Is Expressed in P. falciparum Sporozoites and Plays a Role in Hepatocyte Infection. Cell Rep 22:2951-2963.

52. Vembar SS, Macpherson CR, Sismeiro O, Coppee JY, Scherf A. 2015. The PfAlba1 RNA-binding protein is an important regulator of translational timing in Plasmodium falciparum blood stages. Genome Biol 16:212.

53. Siegel TN, Hon CC, Zhang Q, Lopez-Rubio JJ, Scheidig-Benatar C, Martins RM, Sismeiro O, Coppee JY, Scherf A. 2014. Strand-specific RNA-Seq reveals widespread and developmentally regulated transcription of natural antisense transcripts in Plasmodium falciparum. BMC Genomics 15:150.

54. Martin M. 2011. Cutadapt Removes Adapter Sequences From High-Throughput Sequencing Reads. EMBnet 17:10-12.

55. Pertea M, Kim D, Pertea GM, Leek JT, Salzberg SL. 2016. Transcript-level expression analysis of RNA-seq experiments with HISAT, StringTie and Ballgown. Nat Protoc 11:1650-1667.

56. Li H, Handsaker B, Wysoker A, Fennell T, Ruan J, Homer N, Marth G, Abecasis G, Durbin R. 2009. The Sequence Alignment/Map format and SAMtools. Bioinformatics 25:2078-2079.

57. Liao Y, Smyth GK, Shi W. 2014. featureCounts: an efficient general purpose program for assigning sequence reads to genomic features. Bioinformatics 30:923-930.

58. Ramirez F, Ryan DP, Gruning B, Bhardwaj V, Kilpert F, Richter AS, Heyne S Dundar, F, Manke T. 2016. deepTools2: a next generation web server for deep-sequencing data analysis. Nucleic Aci Res 44:W160-165.

59. Robinson JT, Thorvaldsdottir H, Winckler W, Guttman M, Lander ES, Getz G, Mesirov JP. 2011. Integrative genomics viewer. Nat Biotechnol 29:24-26.

60. Langmead B, Salzberg SL. 2012. Fast gapped-read alignment with Bowtie 2. Nat Methods 9:357-359.

61. Tarasov A, Vilella AJ, Cuppen E, Nijman IJ, Prins P. 2015. Sambamba: fast processing of NGS alignment formats. Bioinformatics 31:2032-2034.

62. Quinlan AR, Hall IM. 2010. BEDTools: a flexible suite of utilities for comparing genomic features. Bioinformatics 26:841-842.

63. Zhang Y, Liu T, Meyer CA, Eeckhoute J, Johnson DS, Bernstein BE, Nusbaum C, Myers RM, Brown M, Li W, Liu XS. 2008. Model-based analysis of ChIP-Seq (MACS). Genome Biol 9:R137.
